# Validation of the Simplified Malaysian Psychosocial Impact of Dental Aesthetics Questionnaire for the Sociodental Approach to Estimate the Orthodontic Treatment Need

**DOI:** 10.3390/ijerph19148665

**Published:** 2022-07-16

**Authors:** Wan Nurazreena Wan Hassan, Mohd Zambri Mohamed Makhbul, Siti Adibah Othman, Zamros Yuzadi Mohd Yusof

**Affiliations:** 1 Department of Paediatric Dentistry and Orthodontics, Faculty of Dentistry, Universiti Malaya, Kuala Lumpur 50603, Malaysia; sitiadibah@um.edu.my; 2 Orthodontic Unit, Klinik Pergigian Cahaya Suria, Pudu Sentral, Ministry of Health Malaysia, Kuala Lumpur 55100, Malaysia; dr.zambri@moh.gov.my; 3 Department of Community Oral Health & Clinical Prevention, Faculty of Dentistry, Universiti Malaya, Kuala Lumpur 50603, Malaysia; zamros@um.edu.my

**Keywords:** sociodental model, short version, index of orthodontic treatment need, malocclusion, validation study, minimal clinical important difference, oral health-related quality of life, adolescent

## Abstract

(1) This study aimed to generate a simplified form of the Malaysian psychosocial impact of dental aesthetics questionnaire (PIDAQ[M]) and validate its use in the sociodental approach for estimating orthodontic treatment need. (2) Two eight-item forms were derived: an impact simplified PIDAQ[M] (ISP8), comprising the most impactful items as rated by 35 participants, and a regression simplified PIDAQ[M] (RSP8), derived from regression analysis of 590 participants’ data from the PIDAQ[M] validation study. Their psychometric performances were assessed for internal consistency, validity (criterion and construct), reproducibility (reliability and agreement), and responsiveness to change. The sociodental estimates were based on 204 orthodontic patients’ data who were assessed for normative need, impact-related need, and propensity-related need. McNemar analysis compared the sociodental estimates when both simplified PIDAQ[M] forms and the original PIDAQ[M] were used to measure impact-related need. (3) Both simplified PIDAQ[M] forms were valid, reproducible, and responsive. The sociodental estimates when using the ISP8 (38.2%) were similar to when the PIDAQ[M] (35.8%) was used (*p* > 0.05) but overestimated by 3.4% (*p* < 0.05) when the RSP8 (39.2%) was used as the assessment tool. (4) The simplified PIDAQ[M] can replace the original PIDAQ[M] in the sociodental approach to estimate the orthodontic treatment needs of the Malaysian population.

## 1. Introduction

The psychosocial impact of the dental aesthetics questionnaire (PIDAQ) is a multidimensional psychometric scale used to measure the impacts of dental arrangement on quality of life on four subscales (psychological impact, PI; social impact, SI; aesthetic concern, AC; and dental self-confidence, DSC). It was developed to support the assessment of orthodontic treatment need [1].

Orthodontic health services at Malaysian government facilities are burdened by the high demand for subsidized treatment. Patients are screened by clinicians for eligibility for orthodontic treatment [2] based on dental health and functional indications using the index of orthodontic treatment need (IOTN), particularly its dental health component (IOTN-DHC) [3]. Recent government guidelines [4] did not adopt the use of its aesthetic component (IOTN-AC), given that such measures of subjective aesthetic impairment [3] have shown disparity in agreement between professionals and subjects [5] and may not justify the distribution of public funds for cosmetic reasons. However, depending on normative need alone, which is an expert-defined need, is inadequate when the determinants for need and demand comprise measures of malocclusion and psychological and social factors [6]. A broader perspective of “need” known as the sociodental approach has been advocated for a more inclusive assessment of orthodontic treatment need [7].

The sociodental model is an algorithm of treatment priority that supports findings of clinical impairments with assessments of the effects of the impairments on patients’ quality of life as well as the chances of having successful treatment outcomes [8]. Patients with normative need, whose problems are supported by impact-related needs and show a good behavioral propensity for successful treatment outcome, would be recommended for orthodontic treatment. Those not fulfilling these three levels of need are proposed for a substitute dental care appropriate to the conditions of their dental health. Such an approach would allow dental service resources to be distributed to patients who require treatment and would benefit most from it [8].

The Malaysian PIDAQ (PIDAQ[M]) is a bilingual validated questionnaire written in the two most frequently used languages in Malaysia, which are Malay [9] and English [10]. The PIDAQ[M] in its current arrangement is lengthy and time-consuming. Patients need to answer 22 items to assess their impact-related need. Therefore, in a busy clinical setting, a simplified form that can capture as much information as the original version may be easier and more practical for use than longer forms that have enhanced psychometric properties [11].

Simplified forms are not uncommon for oral health-related quality of life instruments. They are principally developed with at least two to four items per domain and are used in large epidemiological surveys or clinical settings [12,13]. Simplified forms of the PIDAQ constituting two or three items per domain have recently been shown to be valid and reliable for use by Yemeni adolescents [14].

According to Jokovic et al. [13], the two most common approaches to derive simplified forms are the item impact method and regression method. The item impact method selects items that most impact participants in terms of frequency and severity. The regression method selects items using forward stepwise regression analysis that best predicts the total score of the original instrument.

Thus, this study aimed to generate a simplified PIDAQ[M] form and validate its use in the sociodental model for estimating the orthodontic treatment needs of Malaysian adolescents. In this study, both the item impact and regression methods were applied to determine the most suitable simplified form. Since the short-version Arabic PIDAQ with either two or three items per domain was found to be valid and reliable [14], this study intended to develop the shortest possible form, having only two items per domain. Furthermore, this will also balance the number of items per domain since the AC domain from the original PIDAQ[M] comprised only two items.

The objectives of this study were to (1) assess the validity, reproducibility, and responsiveness to change of the simplified PIDAQ[M] derived using the item impact method and regression method, and (2) to compare the proportions of orthodontic treatment need as identified using the sociodental approach when using the original and simplified PIDAQ[M] as part of its assessment tool. The null hypothesis statement is as follows: a simplified PIDAQ[M] form is not a valid alternative to the original PIDAQ[M] version as part of the assessment tool in the sociodental approach for assessing orthodontic treatment need.

## 2. Materials and Methods

This research was conducted with data collected from a series of studies: Two simplified PIDAQ[M] forms were derived from an item impact study and from data in a psychometric study [9]. The descriptive statistics, validity, and reproducibility of the simplified PIDAQ[M] were examined using cross-sectional data of the psychometric study [9]. The responsiveness to change of the simplified PIDAQ[M] forms was examined using cohort data of patients who had undergone fixed appliance therapy [15]. The use of the simplified PIDAQ[M] compared with the original PIDAQ[M] as applied in the sociodental model was based on data of patients who were screened for orthodontic treatment [16].

### 2.1. Development of the Simplified PIDAQ[M] for Malaysian Adolescents

The simplified PIDAQ[M] was derived using the item impact method and the regression method as recommended by Jokovic et al. [13]. The simplified PIDAQ[M] should reflect most of the information of the original PIDAQ[M]. The sample size for the item impact method based a posteriori [14] was at least 30, while the regression method required at least 163 participants for an anticipated medium effect size (f^2^ = 0.15), 22 predictors, a power level of 0.80, and a probability of 0.05 [17].

#### 2.1.1. Item Impact Method

Thirty-five adolescents with a mean age of 14.7 ± 1.4 years (12 to 17 years old) from the orthodontic treatment waiting list participated in this study by answering a self-administered questionnaire. The questionnaire listed the 22 PIDAQ[M] items [9,10]. Participants who reported to have experienced psychosocial impacts as listed in the 22 PIDAQ[M] items for the past three months were also asked to rate the item importance using a four-point Likert scale from a little bothered (score 1) to extremely bothered (score 4). The item impact score for each item was calculated by multiplying the frequency of adolescents who reported to have psychosocial impacts with the PIDAQ[M] items by the mean rating of the importance of each item. High impact scores indicated high levels of psychosocial impacts, and vice versa. An eight-item simplified form was produced comprising two items with the highest impact scores from each subscale (PI, SI, AC, and DSC). Preliminary analysis showed that three DSC items had similar impact scores (Supplementary Table S1). Therefore, the items with the most frequently reported problems were selected. The finalized form (Table 1) derived using this item impact method will subsequently be referred to as the eight-item impact simplified PIDAQ[M] (ISP8).

#### 2.1.2. Regression Method

Cross-sectional data of 590 secondary school children with a mean age of 14.2 ± 1.5 years (12 to 17 years old) for the psychometric validation of the Malay version of the PIDAQ[M] [9] were used in the regression method. In brief, participants rated their agreement with each of the 22 items using a 5-point Likert scale, ranging from not at all (score 1) to very strongly (score 5). The PIDAQ[M] score was tabulated as the sum of the ratings from the negative PI, SI, and AC domains and reverse scores from the positive DSC domain. A forward stepwise procedure was performed using SPSS. The dependent variable was the PIDAQ[M] score, while the independent variables comprised the scores of the individual items. A model was generated with all the PIDAQ[M] items to identify the most predictable items for the overall score. An 8-item simplified form was generated comprising two items from each subscale (PI, SI, AC, and DSC) that had the highest influence on the coefficient of variation (R^2^). The finalized form (Table 1) derived using this regression method will subsequently be referred to as the eight-item regression simplified PIDAQ[M] (RSP8).

### 2.2. Psychometric Performance of the Simplified PIDAQ

The ISP8 and RSP8 were evaluated for their psychometric performances using the recommended quality criteria [18]: internal consistency, criterion validity, construct validity, reproducibility, and responsiveness to change. The psychometric performance was assessed on the cross-sectional psychometric validation data for the development of the Malay version PIDAQ[M] [9]. Responsiveness to change was assessed using cohort data of 37 adolescents with a mean age of 14.4 ± 2.0 years (11 to 18 years old) who received orthodontic fixed appliance therapy [15]. In both samples, scores of the items were recoded from 0 (not at all) to 4 (very strongly). Scores of each domain were tabulated as the sum of their items. The simplified and original PIDAQ[M] scores were tabulated as the sum of the ratings from their negative PI, SI, and AC domains and reverse scores from the positive DSC domain. Higher simplified or original PIDAQ[M] scores indicated high psychosocial impact, and vice versa.

The sample sizes required were at least 100 or 10 participants per variable for internal consistency, at least 50 participants for construct validity, and at least 50 participants for reproducibility [18]. For responsiveness to change, estimation by G*Power software required at least 10 participants for an anticipated large effect size of 1.0 [15], power of 0.80, and probability of 0.05.

#### 2.2.1. Internal Consistency

Internal consistency was used to assess the extent to which items in the scale measured the same concept [18]. For this, the eight items for each simplified PIDAQ[M] form were assessed for their correlations within the scale. For satisfactory internal consistency, Cronbach’s alpha should be between 0.70 and 0.95 [18], and corrected item–total correlations should be above 0.30 [19].

#### 2.2.2. Validity

Criterion validity was used to assess the extent to which the simplified PIDAQ[M] scores relate to the gold standards, which are instruments that measure impacts caused by malocclusion. For this study, the gold standards were the original PIDAQ[M] and Malay condition-specific Child Oral Impacts on Daily Performances (CS-OIDPc) index [20]. The CS-OIDPc index measures impact on eight daily activities attributed to malocclusion. These activities are the ability to eat, speak, clean their teeth, relax, have emotional stability, smile, do schoolwork, and socialize. The impacts were attributed to malocclusion when participants reported that the impacts were caused by the position of the teeth and gaps between the teeth. The prevalence of CS-OIDPc was recorded if participants reported experiencing problems attributed to their malocclusion in carrying out the eight daily activities in the last three months. The CS-OIDPc performance score was tabulated as the sum of the product of the frequency (scored between 1 and 3) and severity (score between 1 and 3) of problems experienced for each daily activity. A score of “zero” (0) was given if there was no impact attributed to malocclusion.

Construct validity comprises convergent and discriminant validities. The hypothesis to establish convergent validity was that the simplified PIDAQ[M] scores should correlate with participants’ perception of their dental appearance. Their perception was measured using a global scale of self-rating scored from excellent, through good and average, to poor. Discriminant validity was assessed using the index of malocclusion [21]. The index included the IOTN-AC [22] and the awareness component of the perception of occlusal scale (POS-A) [23]. It has a mean value of 0 and was formulated by dividing the total standardized ratings of the IOTN-AC and POS-A by 2. The IOTN-AC comprised subjective ratings from 1 to 10 against matched photographic scales of dental aesthetics, while the POS-A comprised ratings of six occlusal traits using a five-point Likert scale. Both participants and investigators rated the participants’ index of malocclusion, which will subsequently be referred to as MI-S and MI-D, respectively. The hypothesis was that the simplified PIDAQ[M] scores should increase as the index of malocclusion scores increase, and vice versa. Discriminant validity was also assessed using a global measure of patients’ self-assessment of their need for orthodontic treatment. The responses were “need braces”, “did not need braces”, and “unsure” of the need for braces. It was hypothesized that those who felt they needed braces to correct their malocclusion would have higher simplified PIDAQ[M] scores than those who did not think they needed braces.

#### 2.2.3. Reproducibility

Reproducibility, which comprises reliability and agreement, determines the degree of similar answers in repeated measurements [18]. Reliability assesses the degree to which the participants’ scores can be distinguished from each other, while agreement assesses the closeness of the repeated measures [18]. Reproducibility was assessed on 30% of the psychometric validation sample [9] who were recalled two weeks after the first administration. Data of participants who answered the PIDAQ[M] at the first and second administrations were used to determine their simplified PIDAQ[M] scores at the respective time points.

#### 2.2.4. Responsiveness to Change

The simplified PIDAQ[M] forms were assessed for their ability to detect clinically important changes over time. Data of participants who answered the PIDAQ[M] before and after orthodontic treatment were used to determine their simplified PIDAQ[M] scores at the respective time points [15]. The participants also answered a global health transition scale after orthodontic treatment. In this scale, they self-rated their post-treatment dental aesthetics as either “much improved”, “a little improved”, “no difference”, “a little worse”, or “much worse”.

### 2.3. Sociodental Estimates of Orthodontic Treatment Need

**Figure 1:**
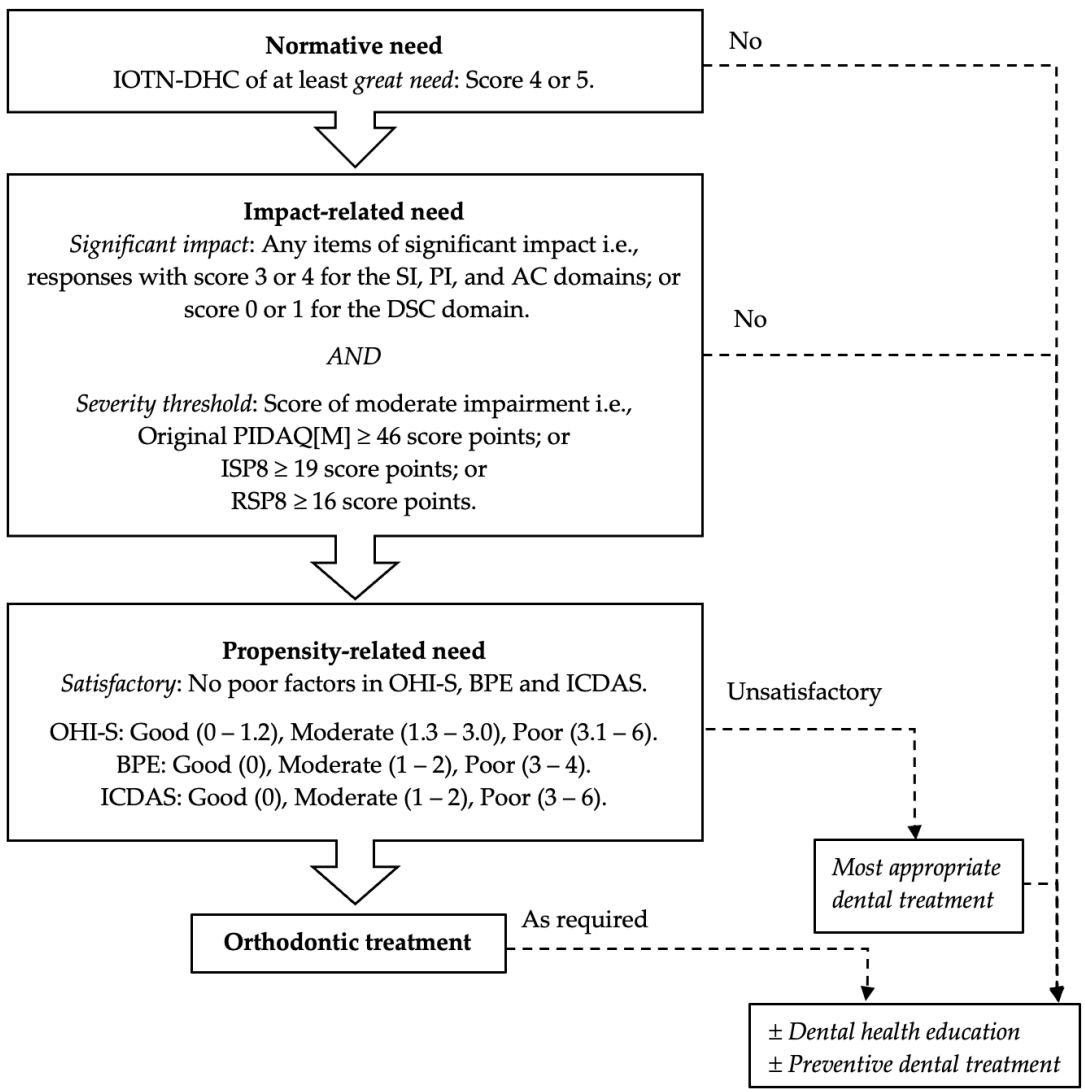
Flowchart of the sociodental approach for assessing orthodontic treatment need.

Data from a cross-sectional study were used to estimate the orthodontic treatment need using the sociodental approach [16]. In brief, 204 adolescents with a mean age of 14.2 ± 1.7 years (11 to 18 years old) who sought orthodontic treatment were screened using the sociodental approach (Figure 1).

First, normative need was assessed by the attending clinicians using the IOTN-DHC. Patients were considered to have normative need when the score was either grade 4 (indicating great need) or grade 5 (indicating very great need) [3].

Second, patients with normative need were then assessed for their impact-related need, as measured using the simplified PIDAQ[M] forms or the 22-item PIDAQ[M] version. Patients were considered to have impact-related need if they experienced significant impact on any of the items and the total score was above a pre-defined threshold for severity. Significant impact items were items of negative domains (PI, SI, and AC) that were rated as score 3 or 4 (indicating agreement with the items) or items of the positive DSC domain that were rated as score 0 or 1 (indicating disagreement with the items). The threshold for the severity score was set at a level considered as moderate impairment, which was acceptably defined as above the 70th percentile of the population norm [24]. Data of Malaysian secondary school children were used to determine the score of the population with self-perceived malocclusion [25]. In brief, the sample comprised 901 subjects with a mean age of 14.1 ± 1.4 years (12 to 17 years old). Of these, 421 who rated themselves using the IOTN-AC with a score of 3 and above (indicating mild, moderate, and severe need) [3] were considered to have self-perceived malocclusion. The severity scores indicating at least moderate impairment were ≥46.0 for the original PIDAQ[M], ≥19.0 for the ISP8, and ≥16.0 for the RSP8.

Third, patients with normative needs and impact-related needs were further assessed for their behavioral propensity to maintain good oral hygiene, periodontal health, and dental health for successful treatment outcome. These were assessed by calibrated examiners [16] using the simplified oral hygiene index (OHI-S) [26], basic periodontal examination (BPE) [27], and international caries detection and assessment system (ICDAS) [28], respectively. These factors were rated as good (OHI-S = 0 to 1.2; BPE = 0; ICDAS = 0), moderate (OHI-S = 1.3 to 3.0; BPE = 1 to 2; ICDAS = 1 to 2), or poor (OHI-S = 3.0 to 6.0; BPE = 3 to 4; ICDAS = 3 to 6) [16]. The propensity-related need was satisfactory when there were no poor factors and unsatisfactory when there was at least one poor factor.

The sociodental approach recommends patients who satisfy all three levels of need to have priority for orthodontic treatment. Patients who do not satisfy any of the needs would have less priority for treatment and are recommended for the most appropriate dental treatment, as well as dental health education and preventive measures [16].

### 2.4. Statistical Analysis

IBM SPSS Statistics 26.0 (IBM Corp, Armonk, NY, USA) and MedCalc^®^ Statistical Software version 20.027 (MedCalc Software Ltd., Ostend, Belgium) were used for data analyses. Descriptive statistics included mean, standard deviation (SD), interquartile values, and range of scores. Floor and ceiling effects of the lowest and highest possible scores, respectively, were recommended to be less than 15% [18].

For criterion validity, the correlations between the simplified PIDAQ[M] and the original PIDAQ[M] were performed using the Spearman correlation. The association of the simplified PIDAQ[M] with the CS-OIDPc prevalence score was analyzed using the Mann–Whitney U test, while the association of the simplified PIDAQ[M] with the CS-OIDPc performance score was analyzed using the Pearson correlation.

Convergent validity was assessed using the association of the simplified PIDAQ[M] with participants’ perception of their dental appearance using the Kruskal–Wallis test. The Mann–Whitney U test compared participants who felt they needed braces against those who did not think they needed braces. Those who were unsure of their need for braces were excluded from the analysis. Discriminant validity, which assessed the association of the simplified PIDAQ[M] with the index of malocclusion, was analyzed using an independent *t*-test.

For reproducibility, the simplified PIDAQ[M] scores between the first and second administrations were analyzed using paired t-test analysis and the intraclass correlation coefficient (ICC) with two-way random effects models for absolute agreement. It is recommended that the paired t-test outcome be insignificant while the ICC be at least 0.7 [18]. The standard error of measurement (SEM) was determined from the square root of the residual variance by analysis of variance (ANOVA). Then, the smallest detectable change (SDC) was derived using the formula SDC = SEM × √2 × 1.96 [18]. Bland–Altman analysis was used to determine the 95% limits of agreement between the two administrations.

In terms of responsiveness, PIDAQ[M] scores were standardized to a scale from 0 to 100. Differences between all three PIDAQ[M] forms (original, ISP8, and RSP8) at pre-treatment and at post-treatment were compared by ANOVA. The minimal clinically important difference (MCID) was determined by the anchor-based approach and distribution approach [29]. In the anchor-based approach, the mean change scores of participants who reported small changes in their OHRQoL following treatment (i.e., “A little improved” and “a little worse”) were first determined. The scores were subtracted by the mean change scores of participants who reported no changes in their OHRQoL. In this study, since none of the participants reported “a little worse” or “no change” following treatment, the mean change score of those who reported a little improvement was used to determine the MCID. This MCID score was supported by the distribution-based approach [29]. In the distribution-based approach, differences in the standardized simplified PIDAQ[M] scores were compared using a paired *t*-test. Their effect size was calculated as differences in the group mean after treatment minus the group mean before treatment and divided by the group standard deviation before treatment [29]. Cohen’s (1988) effect size was interpreted as small (0.2 to <0.5), medium (0.5 to <0.8), or large (≥0.8) [30].

McNemar analysis was used to assess the differences in the proportions of patients with treatment need between normative need and impact-related need, and between normative need and propensity-related need, when either the simplified PIDAQ[M] forms or the original PIDAQ[M] version were used in the assessment of impact-related need. McNemar analysis was also used to compare the differences in the proportions of socio-dental estimates when either the simplified PIDAQ[M] forms or the original PIDAQ[M] version were used in the assessment of impact-related need.

## 3. Results

### 3.1. Content and Descriptive Statistics of the Simplified PIDAQ[M]

Table 1 shows the 12 shortlisted items for the ISP8 and RSP8 derived from the item impact (Supplementary Table S1) and regression methods (Supplementary Table S2), respectively. The ISP8 and RSP8 had four distinct items and shared four common items.

Table 2 shows that the reported scores were slightly higher when using the ISP8 compared to the RSP8. Both forms had neither floor nor ceiling effects (<15%).

The scores standardized to a scale of 0 to 100 showed that the RSP8 had a lower mean score (36.8 ± 22.6) compared to the PIDAQ[M] (40.9 ± 20.2), while the ISP8 had the highest mean score (47.0 ± 20.1). Paired t-tests showed that the mean differences between the original PIDAQ[M] and the simplified PIDAQ[M] forms were statistically significant. The difference with the ISP8 was −6.1 ± 6.8 (*p* < 0.001; 95% CI 5.5 to 6.6), while the difference with the RSP8 was −4.1 ± 6.0 (*p* < 0.001; 95% CI −4.6 to −3.6).

### 3.2. Psychometric Performance of the Simplified PIDAQ[M]

#### 3.2.1. Internal Consistency

The Cronbach alpha scores were 0.85 for the ISP8 and 0.90 for the RSP8. The corrected item–total correlations for both simplified forms were more than 0.30 (Supplementary Table S3).

#### 3.2.2. Criterion Validity

Both simplified PIDAQ[M] forms were almost perfectly correlated with the original PIDAQ[M]. The correlation coefficient of the original PIDAQ[M] with the RSP8 (rho = 0.960) was higher than with the ISP8 (rho = 0.935) (Supplementary Table S4). The ISP8 and RSP8 scores were higher when impacts by malocclusion were prevalent, as measured by CS-OIDPc, than when participants did not report impacts (*p* < 0.05). The simplified PIDAQ[M] scores were also of medium correlation with the CS-OIDPc performance scores (>0.3 and <0.5; *p* < 0.05) (Supplementary Table S5).

#### 3.2.3. Construct Validity

In terms of convergent validity, both simplified PIDAQ[M] forms had statistically significant associations with the self-endorsed dental appearance (*p* < 0.05), with a trend for increasing ISP8 and RSP8 scores as participants rated their teeth from excellent to poor (Supplementary Table S6). Participants who felt that they needed braces also had higher ISP8 and RSP8 scores than those who did not feel that they needed braces (Supplementary Table S7).

In terms of discriminant validity, both simplified PIDAQ[M] forms showed statistically significant differences in the ISP8 and RSP8 scores between participants in the lower and upper quartiles of malocclusion severity as measured using both the MI-S and MI-D (Supplementary Table S8).

#### 3.2.4. Reproducibility

The ICC scores were satisfactorily above 0.70, with the ISP8 having an ICC of 0.84 and the RSP8 having an ICC of 0.90 (*p* < 0.05). The paired t-tests between the repeated measurements were not statistically significant (*p* > 0.05). The ISP8 and RSP8 scores were within the limits of agreement for more than 90% of the participants (Supplementary Table S9).

#### 3.2.5. Responsiveness to Change

The pre-treatment scores standardized to a scale of 0 to 100 were 59.4 ± 20.2 (original PIDAQ[M]), 66.0 ± 19.1 (ISP8), and 59.0 ± 21.2 (RSP8), with differences that were not statistically significant. The post-treatment standardized scores were 29.1 ± 18.1 (original PIDAQ[M]), 31.9 ± 18.4 (ISP8), and 42.1 ± 19.2 (RSP8), which were significantly different. Post hoc pairwise comparison showed that the post-treatment standardized scores of the RSP8 were significantly higher than those of the original PIDAQ[M] by 12.9 (95% CI 2.7 to 23.2; *p* = 0.009). The standardized scores of both the ISP8 and RSP8 demonstrated a significant reduction following orthodontic treatment (*p* < 0.05), with large effect sizes. The ISP8 showed more change (34.0 ± 24.2; ES = 1.7) than the RSP8 (16.9 ± 21.8; ES = 0.8) (Supplementary Table S10).

Both the ISP8 and RSP8 demonstrated a significant reduction following orthodontic treatment (*p* < 0.05) for participants who considered their dental aesthetics as “a little improved” and “much improved”. None of the participants reported no difference or worsening of their dental aesthetics. For the ISP8, participants who reported their dental aesthetics as “much improved” (11.1 ± 7.4) reported slightly more change than those who reported their dental aesthetics as “a little improved” (10.1 ± 9.7). For the RSP8, participants who reported their dental aesthetics as “much improved” (10.7 ± 7.9) reported a similar amount of change compared to participants who reported their dental aesthetics as “a little improved” (10.9 ± 10.1) (Supplementary Table S11).

Table 3 shows that the MCID of the simplified PIDAQ forms was satisfactorily higher than their respective 95% limits of agreement. The MCID for the ISP8, rounded to an integer, was 10 scale points based on the anchor-based approach. The MCID was considered to give a large magnitude of change as supported by an effect size of 1.7 from the distribution-based approach. The MCID for the RSP8, rounded to an integer, was 11 scale points based on the anchor-based approach. The distribution-based approach that supported the magnitude of change was large (ES = 0.8).

### 3.3. Sociodental Estimates of Orthodontic Treatment Need

**Figure 2:**
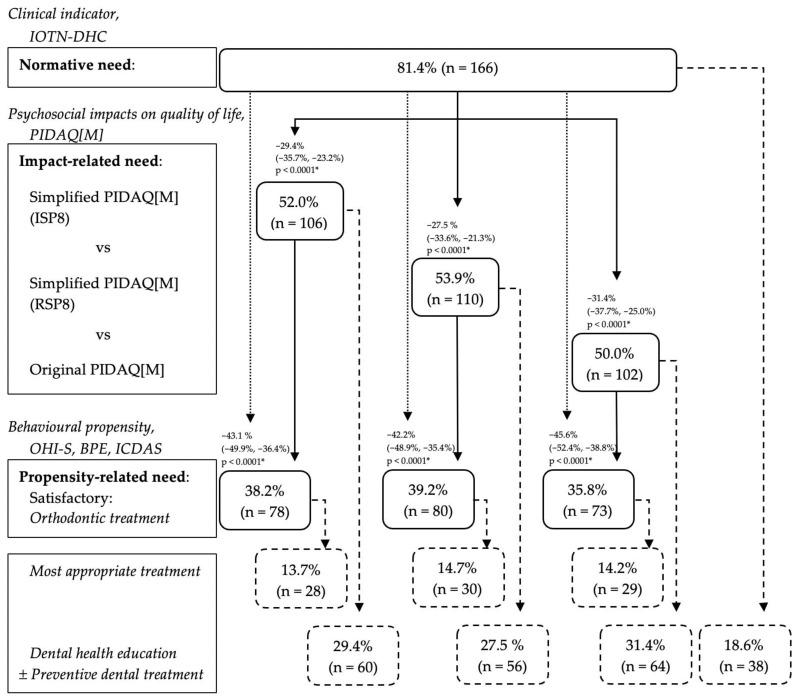
Sociodental approach for assessing orthodontic treatment need comparing the outcomes when either the simplified or the original Malaysian psychosocial impact of dental aesthetics questionnaire (PIDAQ[M]) was used as the impact-related need instrument. Legend: index of orthodontic treatment need, IOTN; simplified oral health index, OHI-S; basic periodontal examination, BPE; index of caries detection and assessment system, ICDAS; no normative need, no impact-related need, or unsatisfactory propensity-related need indicated by a dashed line; * *p* < 0.05, McNemar test.

Figure 2 shows a flowchart of the sociodental approach for estimating orthodontic treatment need. Of the initial 204 patients who requested treatment, 166 (81.4%) had normative need as assessed by the IOTN-DHC. The use of the ISP8 significantly reduced the number of patients who were considered to need treatment (n = 106; 52.0%) by 29.4% (*p* < 0.05). The sociodental approach that considered the normative need, impact-related need by the ISP8, and propensity-related need significantly reduced the number of patients who were considered to need treatment (n = 78; 38.2%) by 43.1% (*p* < 0.05). On the other hand, the use of the RSP8 after normative need assessment reduced the numbers of patients who were considered to need treatment (n = 110; 53.9%) by 27.5% (*p* < 0.05). The sociodental approach that used the RSP8 as the impact-related need tool also showed a reduction in the number of patients who were considered to need treatment (n = 80; 39.2%) by 42.2% (*p* < 0.05). The use of the original PIDAQ[M] had the lowest reduction after normative need assessment (n = 102; 50.0%) of 31.4% (*p* < 0.05), and when the sociodental approach was considered, treatment need was reduced (n = 73; 35.8%) by 45.6% (*p* < 0.05).

Table 4 shows the differences in estimated orthodontic treatment need as assessed by the sociodental approach when using the original PIDAQ[M] as the impact-related need tool compared to the simplified PIDAQ[M]. Compared to the original PIDAQ[M], the difference when using the ISP8 was not significant (2.5%; *p* > 0.05), while the difference when using the RSP8 was small (3.4%; *p* < 0.05).

The difference in the sociodental estimates of orthodontic treatment need when the ISP8 and RSP8 were used in the assessment of impact-related need was not significant (*p* > 0.05) (Table 5).

## 4. Discussion

The simplified PIDAQ[M] forms were derived according to existing shortening methods [12,13,31,32], and their psychometric properties were assessed following recommended criteria [18]. Both simplified PIDAQ[M] forms developed by the item impact and regression methods were valid and reproducible to assess psychosocial impacts by malocclusion among adolescents in Malaysia. When both simplified PIDAQ[M] forms were applied to compare changes following orthodontic treatment, they were also responsive and sensitive to changes in psychosocial impact, indicating that both forms are valid for use as an evaluative tool to assess treatment outcomes. When compared with the original PIDAQ[M], the ISP8 showed no differences in the proportion of patients needing orthodontic treatment as assessed by the sociodental approach, while the difference when using the RSP8 was significant but small.

Recent Malaysian guidelines outlined that treatment shall be accessible to adolescents and adults at government facilities under the Ministry of Health [4] in recognition of the high prevalence of the psychosocial impact of dental aesthetics in adolescents [25] and young adults [33]. However, patients who seek treatment in highly demanded public subsidized health services that screen only by a normative need indicator are usually placed on a waiting list even if they experience significant psychosocial impacts during the waiting period. This is because malocclusion is considered a dental problem that is unlikely to cause adverse health consequences if treatment is deferred [8]. Therefore, an assessment that incorporates evaluation of the impacts of malocclusion such as the socio-dental approach would facilitate prioritization of treatment to the impacted patients who have good behavioral propensities. A simplified PIDAQ[M] that is easy and quick yet able to capture as much information as the full-form PIDAQ[M] for use in the sociodental model is invaluable in a clinical setting. Simplifying questionnaires may also improve response rates [34,35] when used for epidemiological research or in research that aggregates multiple measures per survey.

The simplified PIDAQ[M] forms shared a common item from the DSC and SI domains and both AC items, being the only two items of this domain. Even though they did not share half of the items, their performances were not affected by the differences. Both scales were sensitive as all possible ranges of scores (from 0 to 32) were rated by the participants and had neither floor nor ceiling effects. However, comparisons of the standardized scores indicated that the ISP8 was more sensitive to the impacts experienced by the participants than the original PIDAQ[M], while the RSP8 was the least sensitive. The higher scores in the ISP8 reflect the effect of selecting the higher-valued most frequently bothersome items and removing the lower-valued less frequently bothersome items from the overall score [13]. The RSP8 had smaller differences in the standardized score, a higher Cronbach alpha value, and a higher correlation when compared with the original PIDAQ[M] than the ISP8 because the regression method used to develop the RSP8 selected the best items that predicted the original PIDAQ[M]. Therefore, the RSP8 may be more suitable as an alternative to the original PIDAQ[M] in cross-sectional epidemiological studies because its internal consistency is closer to the original PIDAQ[M].

Criterion validity assessed how well the study instruments relate to the gold standards [18], such as the original PIDAQ[M] or modified constructs to measure the impacts of malocclusion such as the CS-OIDPc [36]. Both simplified forms demonstrated satisfactory associations with these instruments. Construct validity assessed how well the instruments relate to pre-defined hypotheses. In terms of the convergent construct validity, the simplified PIDAQ[M] showed increased scores when participants rated their dental appearance in a decreasing manner from excellent to poor. Those who felt their dental appearance was poor enough to need dental correction had higher scores than those who accepted their dental appearance and did not think that they needed braces. This supports the instruments’ ability to differentiate between good and poor dental aesthetics. In terms of discriminant construct validity, the simplified PIDAQ[M] forms were able to distinguish the severity of malocclusion as measured using indices that measure malocclusion. The evidence supports the instruments’ ability to differentiate between slight and severe malocclusion. Reproducibility is important to ensure the reliability of the instrument to draw similar answers in stable persons [18]. Test–retest results showed that both simplified forms were reliable based on the high ICC and non-significant paired t-test outcomes. In the Bland–Altman analysis, more than 90% of the participants had good agreement between the two test administrations.

The responsiveness of the simplified PIDAQ[M] to detect changes in psychosocial impacts is important for detecting meaningful clinical changes [18]. Both simplified forms were sensitive to change, as their scores reduced significantly with improved dental aesthetics following orthodontic treatment. The change detected by the RSP8 was significantly less than the original PIDAQ[M], while the change detected by the ISP8 was not significantly different to the original PIDAQ[M] post-treatment. The change in the scores of both simplified PIDAQ[M] forms for participants who experienced a small change was higher than the 95% limits of agreement, indicating that they can actually measure the changes that are happening [18]. The MCID is defined as “the smallest change considered as being meaningful by a patient” [29]. The MCIDs determined by the anchor-based approach were similar, with the ISP8 MCID having less than a one-point difference compared to the RSP8. Additionally, the distribution-based approach determined that the magnitude of change detected by both simplified forms was large. The ISP8 showed larger changes than the RSP8 as the items represented what the patients felt was important to them [31]. Therefore, the ISP8 may be more suitable as an alternative to the original PIDAQ[M] in longitudinal clinical studies because its responsiveness was closer to the original PIDAQ[M].

Overall, given that the simplified PIDAQ[M] forms fulfilled the recommended criteria in terms of their measurement properties, the evidence supports both simplified PIDAQ[M] forms as valid, reproducible, and responsive to assess psychosocial impacts due to dental aesthetics.

The sociodental approach would allow orthodontic services and resources to be prioritized as the estimate of treatment need is reduced when compared to using normative need alone [16,37,38]. The current study shows that a normative need threshold based on IOTN-DHC scores of 4 and 5 as set by a local guideline [4] would have allocated treatment to most patients (81.4%) who requested treatment. However, if resources and facilities are not increased to meet the demands for treatment, the waiting time is expected to be longer. This study proposes that the approach for assessing impact-related need should be based not only on patients who are significantly impacted, but also on the severity of the impacts. Therefore, these more stringent criteria will prioritize orthodontic care for those with more severe impacts [8].

In this study, the sociodental estimate of orthodontic treatment need was 35.8% when the original PIDAQ[M] was used as its assessment tool (Figure 2). The proportion of patients who were deemed to need orthodontic treatment as assessed by the sociodental approach was higher with the ISP8 (at 38.2%) and highest with the RSP8 (at 39.2%). The difference in proportions between the simplified forms was not significant. The difference between the ISP8 and the original PIDAQ[M] was also not significant, while the difference between the RSP8 and the original PIDAQ[M] was small (3.4%, *p* < 0.05). Therefore, when choosing a simplified PIDAQ[M] for use in the sociodental approach as an alternative to its original form, the ISP8 is preferred as the difference in proportion was closer to the original PIDAQ[M] compared with the RSP8.

The third level of the sociodental approach also emphasized the concept of need as an ability to benefit. This is based on the premise that patients’ behavioral propensity can affect the treatment outcome and thus should be considered in the assessment of need [39]. In this study, indicators for current oral health conditions were used to reflect the propensity to maintain good oral health. Further research can consider assessing for compliance, which is essential for successful treatment outcomes. This may be assessed using reliable predictors for compliance based on self-perceived smile attractiveness [40] or certain personality traits [41]. These may be more predictable than depending on the severity of malocclusion for predicting compliance [41,42].

The current study demonstrated potential applications of the simplified PIDAQ[M] in the assessment of psychosocial impacts experienced by adolescents. Future research is recommended to assess the cost effectiveness of applying the sociodental approach in orthodontic health services.

### Study Limitations

In this study, the item impact method recruited orthodontic subjects and may not be representative of items that most impact the general adolescent population. Using clinical subjects [13] is considered acceptable since they have expressed need by seeking treatment. Furthermore, it was not feasible to sample the same subjects from the regression method samples for the item impact method, which comprised patients from the orthodontic waiting list and school children—patients who would already have had treatment by this point and experienced changes. In addition, tracing school children was not practical at a time when the Malaysian government enforced various phases of the movement control order to control the chains of COVID-19 disease transmissions. Instead, patients were recruited as clinical service was allowed to continue following precautions [43].

## 5. Conclusions

Within the limitations of this study, the simplified PIDAQ[M] forms for use by Malaysian adolescents derived from the item impact and regression methods were found to be valid, reproducible, and responsive for assessing psychosocial impacts due to dental aesthetics. There was no difference in the sociodental estimates of orthodontic treatment need between the original PIDAQ[M] and the item impact simplified PIDAQ[M] when used as part of the assessment tool, while the difference between the original PIDAQ[M] and the regression simplified PIDAQ[M] was significant but small. Therefore, the simplified PIDAQ[M] can be recommended to replace the original PIDAQ[M] for assessing impact-related need in the sociodental approach for estimating orthodontic treatment need.

## Figures and Tables

**Table 1:** Items of the simplified PIDAQ[M].

Subscale	ISP8 Specific Items	RSP8 Specific Items
**Dental self-confidence**	7. Like to show their teeth 12. Pleased to see own teeth in mirror ^a^	4. Proud of own teeth 12. Pleased to see own teeth in mirror ^a^
**Psychological impact**	11. Others have nicer teeth 20. Wish to look better	6. Distressed because of others’ nice teeth 16. Feel bad about own teeth
**Social impact**	2. Hold back their smile ^a^ 22. Boys/girls find own teeth ugly	2. Hold back their smile ^a^ 9. Teasing
**Aesthetic concern**	1. Don’t like own teeth in mirror ^a^	1. Don’t like own teeth in mirror ^a^
	8. Don’t like own teeth in photos ^a^	8. Don’t like own teeth in photos ^a^

Malaysian psychosocial impact of dental aesthetics questionnaire, PIDAQ[M]; impact simplified PIDAQ[M], ISP8; regression simplified PIDAQ[M], RSP8; ^a^ common item.

**Table 2:** Descriptive statistics for the simplified PIDAQ[M] (n = 590).

Simplified PIDAQ[M]	Mean (SD)	Possible Scores	Range of Scores	Floor (%)	Ceiling (%)	Quartiles		
ISP8	15.1 (6.4)	0–32	0–32	0.5	1.2	11.0	15.0	19.0
RSP8	11.9 (7.2)	0–32	0–32	4.4	0.7	6.0	11.0	16.0

Malaysian psychosocial impact of dental aesthetics questionnaire, PIDAQ[M]; impact simplified PIDAQ[M], ISP8; regression simplified PIDAQ[M], RSP8; standard deviation, SD.

**Table 3:** The MCID of the simplified PIDAQ[M] forms following orthodontic treatment.

	Bland–Altman			MCID		
	95% Limits of Agreement			T1		
**Simplified** **PIDAQ[M]**	Mean	Lower	Upper	^a^ Change in score, mean (SD)	^b^ Standardized effect size (descriptor)	
ISP8	0.4	−7.4	8.2	10.1 (9.7)	1.7	(large)
RSP8	−0.1	−8.8	8.6	10.9 (10.1)	0.8	(large)

Malaysian psychosocial impact of dental aesthetics questionnaire, PIDAQ[M]; impact simplified PIDAQ[M], ISP8; regression simplified PIDAQ[M], RSP8; minimal clinically important difference, MCID; post-treatment, T1; ^a^ anchor-based approach (refer to Table S11); **^b^** distribution-based approach (refer to Table S10); standard deviation, SD.

**Table 4:** Sociodental estimates of orthodontic treatment need when using simplified and original forms of the PIDAQ[M] as the impact-related need instrument (N = 204).

IRN Tool for the Sociodental Approach in Assessing Orthodontic Treatment Need		Original PIDAQ [M]			McNemar Test			
		No PRN	PRN	Total	Diff.	95% CI		*p*-Value
**ISP8**	**No PRN**	125 (61.3%)	1 (0.5%)	126 (61.8%)	−2.5%	−5.0%	0.1%	0.125
	**PRN**	6 (2.9%)	72 (35.3%)	78 (38.2%)				
	**Total**	131 (64.2%)	73 (35.8%)	204				
**RSP8**	**No PRN**	123 (60.3%)	1 (0.5%)	124 (60.8%)	−3.4%	−6.3%	−0.6%	0.039 *
	**PRN**	8 (3.9%)	72 (35.3%)	80 (39.2%)				
	**Total**	131 (57.8%)	73 (35.8%)	204				

Impact-related need, IRN; propensity-related need, PRN; Malaysian psychosocial impact of dental aesthetics questionnaire, PIDAQ[M]; impact simplified PIDAQ[M], ISP8; regression simplified PIDAQ[M], RSP8; difference, diff.; confidence interval, CI; * *p* < 0.05, McNemar test.

**Table 5:** Orthodontic treatment need using the sociodental model as estimated with different versions of the PIDAQ[M] as the impact-related need instrument (N = 204).

IRN Tool for the Sociodental Approach in Assessing Orthodontic Treatment Need		ISP8			McNemar Test			
		No PRN	PRN	Total	Diff.	95% CI		*p*-Value
**RSP8**	**No PRN**	121 (59.3%)	3 (1.5%)	124 (60.8%)	−1.0%	−3.7%	1.7%	0.727
	**PRN**	5 (2.5%)	75 (36.8%)	80 (39.2%)				
	**Total**	126 (61.8%)	78 (38.2%)	204				

Impact-related need, IRN; propensity-related need, PRN; Malaysian psychosocial impact of dental aesthetics questionnaire, PIDAQ[M]; impact simplified PIDAQ[M], ISP8; regression simplified PIDAQ[M], RSP8; difference, diff.; confidence interval, CI; McNemar test.

## Data Availability

The data presented in this study are available on reasonable request from the corresponding author.

## Supplementary Materials

The following supporting information can be downloaded at: https://www.mdpi.com/article/10.3390/ijerph19148665/s1, Table S1: Item impact method showing the impact scores of each PIDAQ[M] item (n = 35); Table S2: Regression method showing items in descending order that contribute to predicting the PIDAQ[M] scores (N = 590); Table S3: Internal consistency: Cronbach’s alpha, scale statistics, and corrected item–total correlations of the simplified PIDAQ[M] forms (n = 590); Table S4: Criterion validity: correlation coefficient between the simplified and original PIDAQ[M] forms (n = 590); Table S5: Criterion validity: comparison of the simplified PIDAQ[M] forms with the CS-OIDP_C_ index (n = 590); Table S6: Convergent validity: comparison of the simplified PIDAQ[M] forms with participants’ perception of the level of their dental appearance (n = 590); Table S7: Convergent validity: comparison of the simplified PIDAQ[M] forms with participants’ perception of their need for braces to correct their dental appearance (n = 590); Table S8: Discriminant validity: comparison of the simplified PIDAQ[M] forms with the severity of malocclusion as rated by the participants (MI-S) and investigators (MI-D) (N = 590); Table S9: Reproducibility of the simplified PIDAQ[M] forms (n = 178); Table S10: Distribution-based approach: changes in standardized simplified PIDAQ[M] scores (N = 37); Table S11: Anchor-based approach: responsiveness of the simplified PIDAQ[M] forms to changes based on the global health transition scale following orthodontic treatment (N = 37).
