# Supplementary material for: Validation of the Simplified Malaysian Psychosocial Impact of Dental Aesthetics Questionnaire for the Sociodental Approach to Estimate the Orthodontic Treatment Need
Source: Int J Environ Res Public Health. 2022 Jul 16;19(14):8665. doi: 10.3390/ijerph19148665 (PMC9324681; doi:10.3390/ijerph19148665)
Supplement: Supplementary file 1 [file ijerph-19-08665-s001.zip › ijerph-1746790-supplementary.pdf]

International Journal of
Environmental Research and Public Health

MDPI

Supplementary Materials:

Table S1. Item impact method showing the impact scores of each PIDAQ[M] item (n= 35).

	Item	Frequency	Importance	Impact
**Dental Self-Confidence**				
4	Proud of own teeth	0.23	1.50	0.34
7	Like to show their teeth [a]	0.20	1.86	0.37
12	Pleased to see own teeth in mirror [a]	0.23	1.63	0.37
17	Teeth look nice to others	0.17	1.33	0.23
21	Satisfied with own teeth's appearance	0.20	1.71	0.34
23	Find own teeth nice	0.17	2.17	0.37
**Psychological Impact**				
3	Envy others for their teeth	0.71	2.32	1.66
6	Distressed because of others' nice teeth	0.71	2.52	1.80
10	Unhappy about own teeth	0.83	2.17	1.80
11	Others have nicer teeth [a]	0.91	2.19	2.00
16	Feel bad about own teeth	0.80	2.32	1.86
20	Wish to look better [a]	0.94	2.70	2.54
**Social Impact**				
2	Hold back their smile [a]	0.86	2.67	2.29
5	What others think	0.63	2.45	1.54
9	Teasing	0.74	2.50	1.86
13	People look strange at my teeth	0.71	2.28	1.63
14	Shy because of own teeth	0.51	2.22	1.14
15	Hiding own teeth	0.51	2.28	1.17
19	Stupid comments from others	0.63	2.50	1.57
22	Boys/Girls find own teeth ugly [a]	0.83	2.59	2.14
**Aesthetic Concern**				
1	Don't like own teeth in mirror [a]	0.80	2.43	1.94
8	Don't like own teeth on photos [a]	0.83	2.55	2.12

[a] Selected items for the item impact method; Malaysian psychosocial impact of dental aesthetics questionnaire, PIDAQ[M].

Table S2. Regression method showing items in descending order that contribute to predicting the PIDAQ[M] scores (N=590).

PIDAQ[M] items				Coefficient of final model				
PI	DSC	SI	AC	Unstandardized coefficients B	t	p-value	95% CI	
							Lower	Upper
6 [a]				.933	11.492	0.000*	.774	1.092
16 [a]				1.158	17.568	0.000*	1.029	1.288
	12 [a]			.992	13.515	0.000*	.848	1.136
		9 [a]		.969	16.440	0.000*	.853	1.085
		2 [a]		1.042	17.792	0.000*	.927	1.157
		19		1.087	23.119	0.000*	.995	1.179
	4 [a]			1.092	16.919	0.000*	.965	1.219
			8 [a]	1.151	18.901	0.000*	1.031	1.270
11				1.058	18.886	0.000*	.948	1.168
	21			1.092	16.847	0.000*	.965	1.219
		5		.972	18.405	0.000*	.868	1.075
		22		1.000	15.759	0.000*	.875	1.125
3				1.041	20.424	0.000*	.941	1.142
		13		1.038	13.509	0.000*	.887	1.189
	7			1.027	20.043	0.000*	.926	1.128
			1 [a]	1.080	16.183	0.000*	.948	1.211
		14		1.184	16.799	0.000*	1.046	1.322
	17			.938	15.060	0.000*	.816	1.061
20				.955	16.801	0.000*	.844	1.067
		15		1.060	16.465	0.000*	.934	1.187
	23			.969	15.648	0.000*	.848	1.091
10				.955	13.454	0.000*	.815	1.094

[a] Selected items for the regression method; Malaysian psychosocial impact of dental aesthetics questionnaire, PIDAQ[M]; psychological impact, PI; dental self-confidence, DSC; social impact, SI; aesthetic concern, AC; confidence interval, CI; *$p < 0.001$.

Table S3. Internal consistency: Cronbach's alpha, scale statistics, and corrected item–total correlations of the simplified PIDAQ[M] forms (n= 590).

Simplified PIDAQ[M]	Cronbach's α	Cronbach's α if item deleted	Scale statistics	Corrected item–total correlation	
			Mean (SD)	Min	Max
ISP8	0.85	0.82-0.84	15.1 (6.4)	0.35	0.67
RSP8	0.90	0.87-0.88	11.8 (7.2)	0.56	0.73

Impact-related need, IRN; propensity-related need, PRN; Malaysian psychosocial impact of dental aesthetics questionnaire, PIDAQ[M]; impact simplified PIDAQ[M], ISP8; regression simplified PIDAQ[M], RSP8; standard deviation, SD.

Table S4. Criterion validity: correlation coefficient between the simplified and original PIDAQ[M] forms (n = 590).

Simplified PIDAQ[M]	Original PIDAQ[M]	
	rho	p-value
ISP8	0.935	0.000*
RSP8	0.960	0.000*

Malaysian psychosocial impact of dental aesthetics questionnaire, PIDAQ[M]; impact simplified PIDAQ[M], ISP8; regression simplified PIDAQ[M], RSP8; Spearman's correlation coefficient, rho; *p < 0.001.

Table S5. Criterion validity: comparison of the simplified PIDAQ[M] forms with the CS-OIDPc index (n=590).

Simplified PIDAQ[M]	CS-OIDPc prevalence	N	Mann–Whitney U						Pearson correlation	
			PIDAQ[M] score					p-value	CS-OIDPc performance	p-value
			Mean	SD	Quartiles					
					Lower	Middle	Upper			
**ISP8**	No	447	13.5	5.7	9.0	14.0	17.0	0.000*	0.461	0.000*
	Yes	143	19.2	6.3	15.0	20.0	24.0			
**RSP8**	No	447	9.9	6.1	5.0	9.0	14.0	0.000*	0.491	0.000*
	Yes	143	17.7	7.3	12.0	18.0	22.0			

Malaysian psychosocial impact of dental aesthetics questionnaire, PIDAQ[M]; condition-specific Child Oral Impacts on Daily Performances, CS-OIDPc; impact simplified PIDAQ[M], ISP8; regression simplified PIDAQ[M], RSP8; standard deviation, SD; *p < 0.001.

Table S6. Convergent validity: comparison of the simplified PIDAQ[M] forms with participants' perception of the level of their dental appearance (n= 590).

Simplified PIDAQ[M]	Rating of dental appearance	N	PIDAQ[M] score					p-value
			Mean	SD	Quartiles			
					Lower	Middle	Upper	
ISP8	Excellent	77	10.0	5.7	6.0	9.0	13.0	0.000*
	Good	283	12.9	5.0	9.0	13.0	16.0	
	Average	182	18.0	4.7	15.0	18.0	21.0	
	Poor	48	24.0	5.9	23.0	25.0	29.0	
RSP8	Excellent	77	6.3	5.8	2.0	5.0	9.0	0.000*
	Good	283	8.9	5.0	6.0	9.0	12.0	
	Average	182	15.5	5.6	12.0	15.5	19.0	
	Poor	48	23.1	7.0	20.0	23.0	28.0	

Malaysian psychosocial impact of dental aesthetics questionnaire, PIDAQ[M]; impact simplified PIDAQ[M], ISP8; regression simplified PIDAQ[M], RSP8; standard deviation, SD; *$p < 0.001$.

Table S7. Convergent validity: comparison of the simplified PIDAQ[M] forms with participants' perception of their need for braces to correct their dental appearance (n= 590).

Simplified PIDAQ[M]	Need braces	N	Simplified PIDAQ[M] scores					p-value
			Mean	SD	Quartiles			
					Lower	Middle	Upper	
ISP8	Yes	247	18.3	6.3	14.0	18.0	23.0	0.000*
	No	200	11.8	5.1	8.0	12.0	15.8	
RSP8	Yes	247	15.9	7.2	10.0	16.0	21.0	0.000*
	No	200	8.0	5.1	4.0	8.0	11.0	

Malaysian psychosocial impact of dental aesthetics questionnaire, PIDAQ[M]; impact simplified PIDAQ[M], ISP8; regression simplified PIDAQ[M], RSP8; standard deviation, SD; *$p < 0.001$.

Table S8. Discriminant validity: comparison of the simplified PIDAQ[M] forms with the severity of malocclusion as rated by the participants (MI-S) and investigators (MI-D) (= 590).

	MI-S	Quartile			
		Lower	Upper		
	N	147	147		
Simplified PIDAQ[M]		Mean (SD)	Mean (SD)	Effect size	p-value
ISP8		10.6 (5.4)	20.0 (5.7)	1.70	0.000*
RSP8		6.5 (5.4)	17.8 (6.7)	1.89	0.000*
	**MI-D**	**Quartile**			
		**Lower**	**Upper**		
	**N**	147	148		
**Simplified PIDAQ[M]**		**Mean (SD)**	**Mean (SD)**	**Effect size**	**p-value**
ISP8		12.6 (5.6)	17.1 (5.6)	0.74	0.000*
RSP8		8.6 (6.1)	14.5 (7.3)	0.88	0.000*

Self-rated malocclusion index, MI-S; investigator-rated malocclusion index, MI-D; Malaysian psychosocial impact of dental aesthetics questionnaire, PIDAQ[M]; impact simplified PIDAQ[M], ISP8; regression simplified PIDAQ[M], RSP8; standard deviation, SD; *p < 0.001.

Table S9. Reproducibility of the simplified PIDAQ[M] forms (n=178).

Simplified PIDAQ[M]	$ICC_{agreement}$ (95% CI)	SEM	SDC	Paired t-test Diff	(SD)	Bland–Altman 95% limits of agreement Lower	Upper	Within limits (%)
ISP8	0.84 (0.79-0.88)*	2.81	7.78	0.38	3.97	-7.40	8.16	94.4
RSP8	0.90 (0.87-0.93)*	3.13	8.67	-0.12	4.42	-8.78	8.55	92.1

Malaysian psychosocial impact of dental aesthetics questionnaire, PIDAQ[M]; impact simplified PIDAQ[M], ISP8; regression simplified PIDAQ[M], RSP8; *p < 0.05; confidence interval, CI; standard error of measurement, SEM; smallest detectable change, SDC; mean differences, Diff; standard deviation, SD.

Table S10. Distribution-based approach: changes in standardized simplified PIDAQ[M] scores (N=37).

	Standardized score		Change in standardized score					
	T0	T1	T1-T0					
Simplified PIDAQ[M]	Mean (SD)	Mean (SD)	*Mean change (SD)*	*95% CI Lower*	*95% CI Upper*	*p-value*	*Standardized effect size*	*Effect size descriptor*
**ISP8**	66.0 (19.9)	31.9 (18.4)	34.0 (24.2)	26.0	42.1	.000*	1.7	large
**RSP8**	59.0 (21.3)	42.1 (19.2)	16.9 (21.8)	9.6	24.2	.000*	0.8	large

Malaysian psychosocial impact of dental aesthetics questionnaire, PIDAQ[M]; impact simplified PIDAQ[M], ISP8; regression simplified PIDAQ[M], RSP8; pre-treatment, T0; post-treatment, T1; standard deviation, SD; confidence interval, CI; *p < 0.001.

Table S11. Anchor-based approach: responsiveness of the simplified PIDAQ[M] forms to changes based on the global health transition scale following orthodontic treatment (N=37).

Global health transition scale	Simplified PIDAQ[M]	Responsiveness to change at T1						
		n	T0 Mean (SD)	T1 Mean (SD)	p-value	Change in score, mean (SD)	Effect size d	Effect size Descriptor
**A little improved**	**ISP8**	7	20.6 (7.1)	10.4 (4.0)	.033*	10.1 (9.7)	1.8	Large
	**RSP8**		18.7 (7.9)	7.9 (4.9)	.029*	10.9 (10.1)	1.6	Large
**Much improved**	**ISP8**	30	21.2 (6.0)	10.2 (6.3)	.000*	11.1 (7.4)	1.8	Large
	**RSP8**		18.9 (6.7)	8.2 (6.9)	.000*	10.7 (7.9)	1.6	Large

Malaysian psychosocial impact of dental aesthetics questionnaire, PIDAQ[M]; impact simplified PIDAQ[M], ISP8; regression simplified PIDAQ[M], RSP8; pre-treatment, T0; post-treatment, T1; standard deviation, SD; confidence interval, CI; *p < 0.001.
